# Risk of contralateral breast cancer according to first breast cancer characteristics among women in the USA, 1992–2016

**DOI:** 10.1186/s13058-021-01400-3

**Published:** 2021-02-17

**Authors:** Cody Ramin, Diana R. Withrow, Brittny C. Davis Lynn, Gretchen L. Gierach, Amy Berrington de González

**Affiliations:** 1 Radiation Epidemiology Branch, Division of Cancer Epidemiology and Genetics, National Cancer Institute, National Institutes of Health, Bethesda, MD USA; 2 Integrative Tumor Epidemiology Branch, Division of Cancer Epidemiology and Genetics, National Cancer Institute, National Institutes of Health, Bethesda, MD USA

**Keywords:** Breast cancer, Contralateral breast cancer, SEER

## Abstract

**Background:**

Estimates of contralateral breast cancer (CBC) risk in the modern treatment era by year of diagnosis and characteristics of the first breast cancer are needed to assess the impact of recent advances in breast cancer treatment and inform clinical decision making.

**Methods:**

We examined CBC risk among 419,818 women (age 30–84 years) who were diagnosed with a first unilateral invasive breast cancer and survived ≥ 1 year in the US Surveillance, Epidemiology, and End Results program cancer registries from 1992 to 2015 (follow-up through 2016). CBC was defined as a second invasive breast cancer in the contralateral breast ≥ 12 months after the first breast cancer. We estimated standardized incidence ratios (SIRs) of CBC by year of diagnosis, age at diagnosis, and tumor characteristics for the first breast cancer. Cumulative incidence of CBC was calculated for women diagnosed with a first breast cancer in the recent treatment era (2004–2015, follow-up through 2016).

**Results:**

Over a median follow-up of 8 years (range 1–25 years), 12,986 breast cancer patients developed CBC. Overall, breast cancer patients had approximately twice the risk of developing cancer in the contralateral breast when compared to that expected in the general population (SIR = 2.21, 95% CI = 2.17–2.25). SIRs for CBC declined by year of first diagnosis, irrespective of age at diagnosis and estrogen receptor (ER) status (*p*-trends < 0.001), but the strongest decline was after an ER-positive tumor. The 5-year cumulative incidence of CBC ranged from 1.01% (95% CI = 0.90–1.14%) in younger women (age < 50 years) with a first ER-positive tumor to 1.89% (95% CI = 1.61–2.21%) in younger women with a first ER-negative tumor.

**Conclusion:**

Declines in CBC risk are consistent with continued advances in breast cancer treatment. The updated estimates of cumulative incidence inform breast cancer patients and clinicians on the risk of CBC and may help guide treatment decisions.

**Supplementary Information:**

The online version contains supplementary material available at 10.1186/s13058-021-01400-3.

## Background

In the USA, overall 5-year survival rates for breast cancer now exceed 90% [1] and a growing number of breast cancer patients will become long-term survivors at risk for second cancers. Contralateral breast cancer (CBC) is the most common second cancer among breast cancer patients [2]. Although the number of women at risk for CBC is increasing, previous studies have reported an overall decrease in CBC incidence rates since the 1980s [3, 4]. Specifically, CBC incidence has declined annually by 3% from 1985 through 2006 in the USA [4]. Importantly, approaches to breast cancer treatment have evolved over the past several decades with the widespread uptake of adjuvant hormone therapy, including tamoxifen in the 1990s and aromatase inhibitors in the mid-2000s, advances in chemotherapy regimens including a shift towards taxane-containing drugs [5–7], and the introduction of trastuzumab to treat early-stage human epidermal growth factor receptor 2 (HER2)-positive tumors in 2005 [8, 9]. In addition, the uptake of contralateral prophylactic mastectomies in the USA has nearly tripled in the past decade to over 10% of invasive breast cancer patients undergoing the removal of the unaffected breast [10–12]. This increasing uptake of contralateral prophylactic mastectomies has occurred across all ages and stage at diagnosis [11, 12].

Thus, the aim of our study was to describe CBC risk in the USA across these periods of significant advances in treatment and to include assessment by HER2 status for the first time as well as immunohistochemistry (IHC)-defined breast cancer subtypes. For this study, we used nationally representative data from the National Cancer Institute’s Surveillance, Epidemiology, and End Results (SEER) program, a large-scale and population-based group of cancer registries with detailed records and long-term follow up, to examine CBC risk compared to the general population from 1992 to 2016 according to calendar year of diagnosis, age at diagnosis, and tumor characteristics for the first breast cancer. To help inform clinical decision making, we also calculated the 5-year cumulative incidence of CBC in the recent treatment era from 2004 to 2016 according to characteristics of the first breast cancer.

## Methods

### Study population

We obtained female breast cancer case and population data using the SEER 13 Registries Database excluding the Alaska Native Tumor Registry (November 2018 submission, 1992–2016) [13]. We used the SEER 13 Registries Database because it includes the time periods when there were major advances in breast cancer treatment and provides long-term follow-up to examine trends in CBC risk. We identified women aged 30 to 84 years who were diagnosed with a first primary unilateral invasive breast cancer with known laterality and who survived ≥ 1 year without developing a second cancer. We excluded women aged < 30 years given the relatively low number of breast cancers diagnosed prior to this age and women aged ≥ 85 years due to under-reporting of second cancers among older patients [14]. Those diagnosed with a first breast cancer at stage IV or based on autopsy reports/death certificates only were not included. The analytic cohort included 419,818 women with invasive unilateral breast cancer who survived for ≥ 1 year and who were diagnosed between 1992 and 2015 and followed through 2016.

### Outcome assessment

We defined CBC as an invasive second breast cancer diagnosed in the contralateral unaffected breast ≥ 12 months after the first invasive breast cancer diagnosis. We excluded the first 12 months of follow-up to reduce potential misclassification of metastases or undetected synchronous bilateral breast cancer as CBC [4]. Follow-up time started 12 months after the diagnosis of the first breast cancer and continued until the second cancer diagnosis, death, loss to follow-up, or administrative censoring on December 31, 2016, whichever occurred first.

### Covariates

Patient and tumor characteristics for the first breast cancer included calendar year of diagnosis, age at diagnosis, American Joint Committee on Cancer (AJCC) stage (I, II, III, IV), histology (ductal, lobular, mixed, other), estrogen receptor (ER) status (positive, negative, borderline, or unknown), HER2 status (positive, negative, borderline, or unknown), and IHC-defined breast cancer subtype (hormone receptor [HR]+/HER2+, HR+/HER2−, HR−/HER2+, HR−/HER2− [triple negative], or unknown). HR status was defined as a joint combination of ER and progesterone receptor (PR) status (i.e., HR+ = ER+ and/or PR+; HR− = ER− and PR−). For breast cancer subtype, borderline tumors were categorized as unknown for HER2 status and positive for ER/PR status using the standard SEER algorithm. ER/PR status has been routinely collected in the SEER database since 1990 while collection of HER2 status began in 2010. Histologic categories were based on the International Classification of Diseases for Oncology, third edition (ICD-O-3) codes, and categorized as ductal (8500, 8523), lobular (8520, 8524), mixed ductal/lobular (8522), and other (all remaining codes). First course of treatment (radiation, chemotherapy, and hormone therapy) has been recorded in the SEER registries and categorized as known receipt (yes) and no or unknown receipt (no/unknown). Women without treatment and unknown treatment are combined in SEER data due to the limited sensitivity of treatment variables in the SEER registries [15]. Type of breast cancer surgery has been routinely recorded since 1998. We defined breast cancer surgery as none, breast conserving therapy, unilateral mastectomy, contralateral prophylactic mastectomy, or unknown. Contralateral prophylactic mastectomy was defined as the removal of the affected breast and the uninvolved contralateral breast.

### Statistical analysis

We estimated standardized incidence ratios (SIRs, a measure of relative risk) and 95% confidence intervals (CIs) for CBC overall and according to characteristics of the first breast cancer. SIRs for CBC by HER2 status and subtype of the first breast cancer were also evaluated in a subgroup of women diagnosed with a first breast cancer between 2010 and 2015 and followed through 2016. SIRs were calculated as the observed number of incident breast cancers (in the contralateral breast) compared to the expected number in the general population [16]. Expected breast cancers were derived from incidence rates in the 12 SEER registries stratified by race (White/unknown, Black, other), age at initial diagnosis (5-year groups), and calendar year (5-year intervals) [16]. We estimated 95% confidence intervals using the Byar’s approximation of the exact Poisson distribution [17].

To describe temporal trends in CBC risk, SIRs for CBC were stratified by calendar period of first breast cancer diagnosis (1992–1997, 1998–2003, 2004–2009, 2010–2015). We first conducted this analysis including all women to describe long-term trends in CBC risk among the general population of breast cancer survivors. We then estimated SIRs excluding patients with contralateral prophylactic mastectomies or unknown surgery type among those diagnosed with a first breast cancer from 1998+ when type of breast cancer surgery was available in SEER. We did not exclude these patients in our main approach since we aimed to describe CBC risk on a population level and found that exclusion of women with contralateral prophylactic mastectomies did not substantially change our findings. Trends were examined overall and according to age at diagnosis (< 50 years and ≥ 50 years, as a proxy for menopausal status [18]) and ER status for the initial breast cancer. Trends by detailed categories of age at diagnosis (< 40, 40 to < 50, 50 to < 60, 60 to < 70, ≥ 70 years) for the first breast cancer were also described. Exploratory analyses examined trends by initial receipt of chemotherapy and hormone therapy. We also conducted several sensitivity analyses. First, we examined trends by time since first breast cancer diagnosis (< 5 years, ≥ 5 years). Second, we expanded CBC to include both in situ and invasive tumors. Third, we varied the definition of CBC to those diagnosed ≥ 6 and ≥ 24 months after the first breast cancer.

To provide an absolute measure of CBC risk in the recent treatment era, we estimated cumulative incidence of CBC among 1-year survivors diagnosed with a first breast cancer between 2004 and 2015 and followed through 2016. We estimated cumulative incidence of CBC overall and according to characteristics of the first breast cancer. We calculated the 5-year cumulative incidence of CBC from the index date using a competing risk approach to account for death and other second cancers as competing events [19, 20]. We excluded women with contralateral prophylactic mastectomies or unknown surgery type from all estimates of cumulative incidence. As a sensitivity analysis, we defined CBC as those diagnosed ≥ 6 months after the first breast cancer to estimate the impact of earlier detection.

All statistical tests were two-sided and *p* values < 0.05 were considered statistically significant. *P* for trends were calculated using the smrby package in Stata. Analyses were performed using SEER*Stat software (version 8.3.5), Stata version 11 and 15 (StataCorp, College Station, TX, USA).

## Results

Table 1 describes characteristics of the first breast cancer overall and by calendar period of diagnosis among 419,818 1-year survivors with a median follow-up of 7.9 years (range 1.00 – 24.9 years). The mean age at first breast cancer diagnosis was 59 years and tumors were primarily early stage, ductal histology, and ER-positive. Characteristics of the first breast cancer were generally similar across calendar period of diagnosis. However, the proportion of first ER-positive tumors increased over time. From 1998 to 2003 and 2010 to 2015, the uptake of contralateral prophylactic mastectomies more than tripled from 2.9 to 11.8% of breast cancer patients, while the proportion of women undergoing breast conserving surgery or unilateral mastectomy decreased from 91.3 to 83.4%.

**Table 1:** Descriptive characteristics of the first breast cancer among 1-year survivors of a first primary breast cancer in 12 SEER registries, 1992–2016

		Calendar period of first breast cancer diagnosis			
	Overall	1992–1997	1998–2003	2004–2009	2010–2015
Characteristics of the first breast cancer	*N* = 419,818	*N* = 89,052	*N* = 107,103	N = 107,828	*N* = 115,835
Overall, %	100.0	21.2	25.5	25.7	27.6
Year of diagnosis, mean years (SD)	2004.0 (6.8)	1994.6 (1.7)	2000.5 (1.7)	2006.5 (1.7)	2012.5 (1.7)
Age at diagnosis, years, %					
< 40	5.9	6.7	6.1	5.8	5.3
40 to < 50	20.4	20.7	20.8	21.4	18.8
50 to < 60	26.1	23.0	26.7	27.5	26.6
60 to < 70	24.3	23.4	21.9	23.6	27.9
≥ 70	23.3	26.2	24.6	21.7	21.4
Age at diagnosis, mean years (SD)	58.8 (12.7)	59.2 (13.1)	58.8 (12.9)	58.4 (12.6)	59.0 (12.2)
Stage at diagnosis, %					
I	50.7	50.9	50.9	50.2	50.7
II	36.0	33.8	35.4	36.7	37.7
III	13.3	15.4	13.7	13.1	11.6
Histology, %					
Ductal	75.4	73.4	72.3	76.3	79.1
Lobular	8.3	7.7	8.1	8.1	9.3
Mixed	7.1	5.7	9.1	7.8	5.6
Other	9.2	13.3	10.5	7.8	6.1
ER status, %					
Positive	73.1	62.0	68.6	76.4	82.8
Negative	18.6	20.2	18.6	19.8	16.0
Borderline/unknown	8.3	17.7	12.8	3.7	1.2
Initial treatment receipt, %					
Surgery^a^					
None	2.0	–	1.2	1.7	3.1
BCS/unilateral mastectomy	87.9	–	91.3	89.5	83.4
Contralateral prophylactic mastectomy	7.3	–	2.9	6.8	11.8
Unknown	2.8	–	4.6	2.0	1.7
Radiation therapy					
Yes	54.2	45.0	55.3	56.8	58.0
No/unknown	45.8	55.1	44.7	43.2	42.1
Chemotherapy					
Yes	40.5	31.3	41.9	45.0	42.0
No/unknown	59.5	68.8	58.1	55.0	58.0
Hormone therapy					
Yes	42.9	32.5	37.4	43.2	55.6
No/unknown	57.1	67.5	62.6	56.8	44.4
HER2 status^b^, %					
Positive	14.5	–	–	–	14.5
Negative	80.6	–	–	–	80.6
Borderline/unknown	4.9	–	–	–	4.9
Breast cancer subtype^b,c^, %					
HR+/HER2+	10.2	–	–	–	10.2
HR+/HER2−	70.3	–	–	–	70.3
HR−/HER2+	4.3	–	–	–	4.3
HR−/HER2− (Triple negative)	10.2	–	–	–	10.2
Unknown	5.0	–	–	–	5.0

*SEER*, Surveillance, Epidemiology, and End Results; *ER*, estrogen receptor; *BCS*, breast conserving surgery; *HER2*, human epidermal growth factor receptor 2; *HR*, hormone receptor; *PR*, progesterone receptor

^a^Information on initial surgery was available for breast cancers diagnosed from 1998+ (*N* = 330,766)

^b^HER2 status was routinely collected in 2010+. Estimates include first breast cancers diagnosed from 2010 to 2015 and followed through 2016 (*N* = 115,835)

^c^HR+ = ER+ and/or PR+; HR− = ER− and PR−

**Fig. 1:**
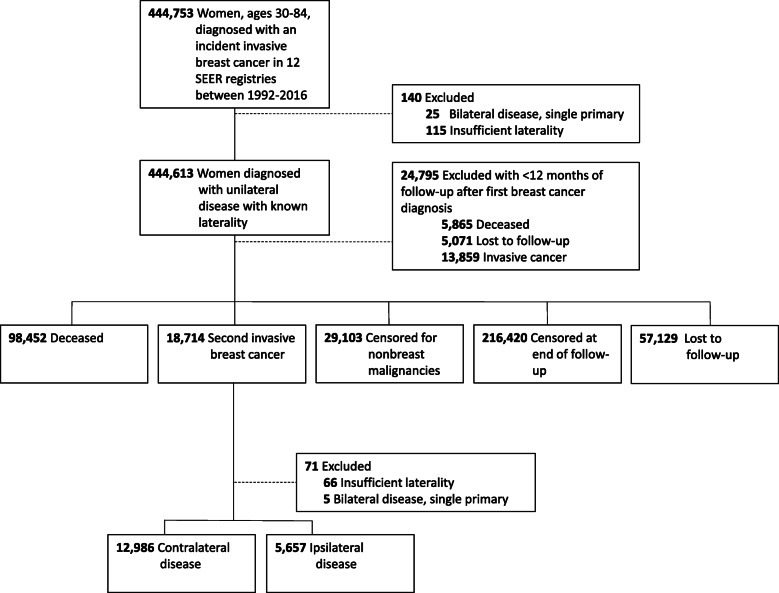
Criteria for defining contralateral breast cancer cases among women diagnosed with a first breast cancer between 1992 and 2015 and followed through 2016 in 12 SEER registries

We identified 12,986 cases of CBC among women diagnosed with a first breast cancer between 1992 and 2015 and followed through 2016 (Fig. 1). Breast cancer patients had approximately a 2-fold increased risk of an incident breast cancer (in the contralateral breast) when compared to that expected in the general population (SIR = 2.21, 95% CI = 2.17–2.25) (Table 2). SIRs for CBC were highest among women diagnosed with a first breast cancer at age < 40 years (SIR = 6.41, 95% CI = 6.02–6.82), and after a first ER-negative tumor (SIR = 3.05; 95% CI = 2.94–3.16). CBC risk increased from over twofold in women diagnosed at stage I (SIR = 2.08, 95% CI = 2.03–2.13) to nearly threefold in those diagnosed at stage III (SIR = 2.97, 95% CI = 2.83–3.11). SIRs by histologic type of the first breast cancer ranged between 2.06 and 2.49 with the highest SIR observed among mixed ductal/lobular histology. SIRs were approximately 2-fold regardless of time since diagnosis with a slightly higher SIR observed with longer latency (< 5 years: SIR = 2.03, 95% CI = 1.97–2.09; ≥ 5 years: SIR = 2.32, 95% CI = 2.27–2.37). Compared to the overall SIR of 2.21, SIRs for CBC were slightly higher among women treated with chemotherapy (SIR = 2.49, 95% CI = 2.42–2.56) and lower among women with hormone therapy (SIR = 1.88, 95% CI = 1.83–1.94).

**Table 2:** SIRs for contralateral breast cancer among 1-year survivors of a first primary breast cancer in 12 SEER registries, 1992–2016

First breast cancer characteristic	No. of CBCs (%)	SIR (95% CI)
Overall	12,986 (100.0)	2.21 (2.17 to 2.25)
Age at diagnosis, years		
< 40	1002 (7.7)	6.41 (6.02 to 6.82)
40 to < 50	2691 (20.7)	2.79 (2.69 to 2.90)
50 to < 60	3452 (26.6)	2.07 (2.00 to 2.14)
60 to < 70	3292 (25.4)	1.91 (1.85 to 1.98)
≥ 70	2549 (19.6)	1.87 (1.80 to 1.94)
Time since diagnosis		
< 5 years	4620 (35.6)	2.03 (1.97 to 2.09)
≥ 5 years	8366 (64.4)	2.32 (2.27 to 2.37)
Stage at diagnosis		
I	6899 (53.1)	2.08 (2.03 to 2.13)
II	4364 (33.6)	2.21 (2.14 to 2.28)
III	1723 (13.3)	2.97 (2.83 to 3.11)
Histology		
Ductal	9340 (71.9)	2.17 (2.13 to 2.21)
Lobular	1037 (8.0)	2.06 (1.94 to 2.19)
Mixed	1126 (8.7)	2.49 (2.35 to 2.64)
Other	1483 (11.4)	2.44 (2.32 to 2.57)
ER status		
Positive	8372 (64.5)	1.98 (1.94 to 2.02)
Negative	3024 (23.3)	3.05 (2.94 to 3.16)
Initial treatment		
Radiation therapy		
Yes	7180 (55.3)	2.25 (2.20 to 2.30)
No/unknown	5806 (44.7)	2.17 (2.11 to 2.23)
Chemotherapy		
Yes	5086 (39.2)	2.49 (2.42 to 2.56)
No/unknown	7900 (60.8)	2.06 (2.01 to 2.11)
Hormone therapy		
Yes	4442 (34.2)	1.88 (1.83 to 1.94)
No/unknown	8544 (65.8)	2.43 (2.38 to 2.48)
HER2 status^a^		
Positive	93 (11.8)	1.45 (1.17 to 1.78)
Negative	646 (82.2)	1.57 (1.45 to 1.70)
Breast cancer subtype^a,b^		
HR+/HER2+	66 (8.4)	1.47 (1.14 to 1.87)
HR+/HER2−	534 (67.9)	1.47 (1.35 to 1.60)
HR−/HER2+	27 (3.4)	1.42 (0.94 to 2.07)
HR−/HER2− (triple negative)	111 (14.1)	2.43 (2.00 to 2.93)

*SEER*, Surveillance, Epidemiology, and End Results; *CBC*, contralateral breast cancer; *SIRs*, standardized incidence ratios; *CI*, confidence interval; *ER*, estrogen receptor; *HER2*, human epidermal growth factor receptor 2; *HR*, hormone receptor; *PR*, progesterone receptor

^a^HER2 status routinely collected in 2010 and onwards. Estimates include first breast cancers diagnosed between 2010 and 2015 and followed through 2016 (no. of first breast cancers = 115,835; no. of contralateral breast cancers = 786; overall SIR = 1.57, 95% CI = 1.46–1.68)

^b^HR+ = ER+ and/or PR+; HR− = ER− and PR−

In the subset of women diagnosed with a first breast cancer between 2010 and 2015 and followed through 2016 (overall SIR = 1.57; 95% CI = 1.46–1.68; Table 2), SIRs for CBC were similar by HER2 status of the first breast cancer (HER2-positive: SIR = 1.45, 95% CI = 1.17–1.78; HER2-negative: SIR = 1.57, 95% CI = 1.45–1.70). CBC risk was highest after a first triple-negative breast cancer (SIR = 2.43, 95% CI = 2.00–2.93; SIRs for the other subtypes ranged from 1.42 to 1.47).

**Fig. 2:**
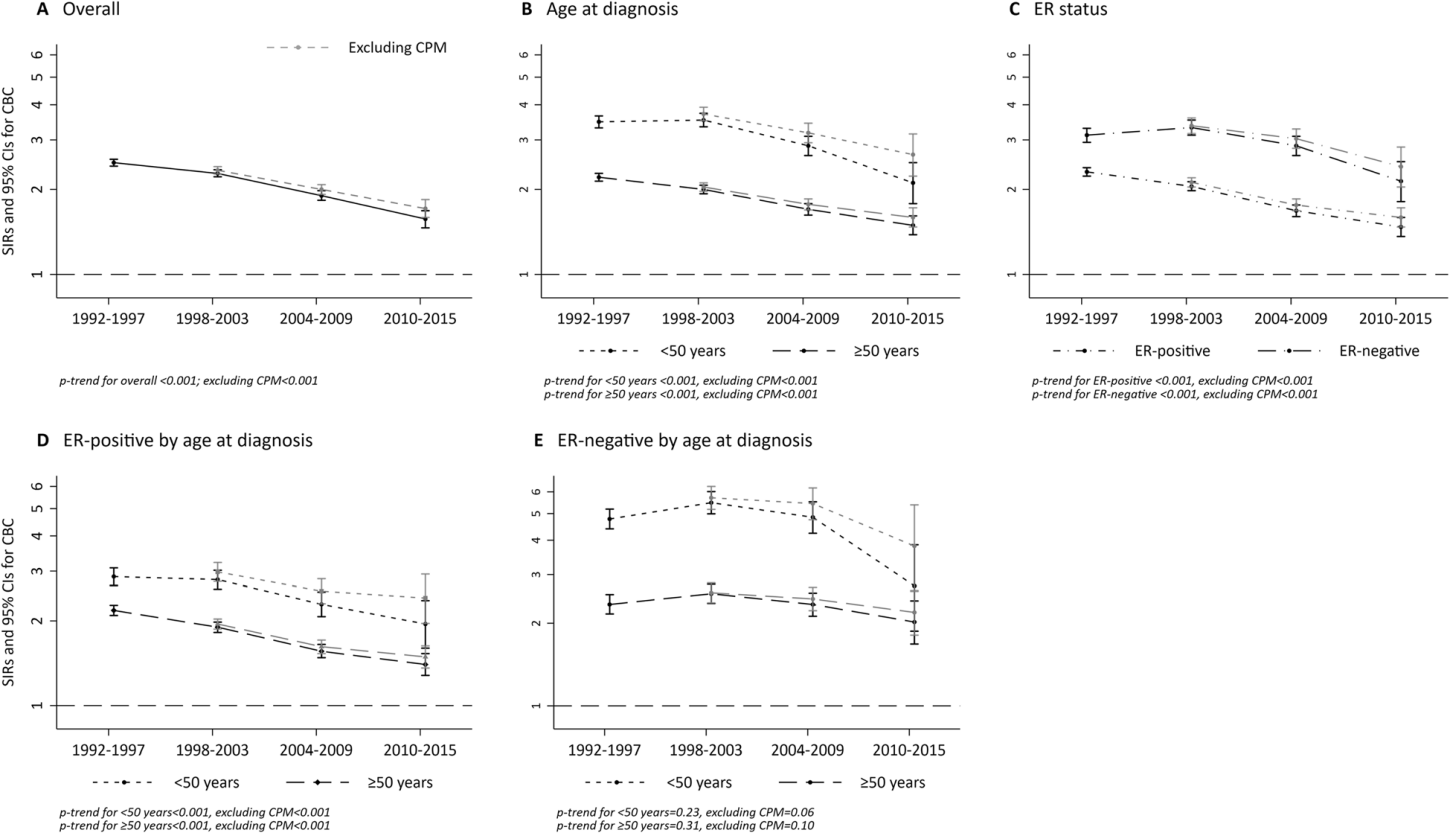
Temporal trends in SIRs for contralateral breast cancer overall and excluding women with contralateral prophylactic mastectomy (CPM) among 1-year survivors of a first primary breast cancer in 12 SEER registries, 1992–2016. Estimates for contralateral breast cancer by calendar period of first breast cancer diagnosis are **a** overall, and according to **b** age at first breast cancer diagnosis, **c** ER status of first breast cancer, **d** after ER-positive breast cancer by age at first diagnosis, and **e** after ER-negative breast cancer by age at first diagnosis. Gray dashed lines indicate exclusion of CPM

SIRs for CBC significantly declined over calendar period of diagnosis (Fig. 2; Additional file 1: Supplementary Table 1). The overall SIR for CBC decreased from 2.49 (95% CI = 2.42–2.56) in 1992–1997 to 1.57 (95% CI = 1.46–1.68) in 2010-2015 (*p*-trend < 0.001). This decreasing temporal trend in CBC risk was observed regardless of age at diagnosis and ER status of the first breast cancer (*p*-trends < 0.001). Trends in CBC risk differed with joint stratification by age at diagnosis and ER status of the first breast cancer. Specifically, we observed a significant decreasing trend in CBC risk regardless of age at diagnosis after an ER-positive tumor (*p*-trends < 0.001) but not after an ER-negative tumor (*p*-trend for < 50 years = 0.23; *p*-trend for ≥ 50 years = 0.31). The observed overall decline in CBC risk after an ER-negative tumor appeared driven by younger women diagnosed with a first breast cancer between 2010 and 2015. SIRs were overall slightly higher in magnitude with exclusion of women with contralateral prophylactic mastectomies, but similar temporal trends were observed even with the exclusion of these patients. Trends by detailed categories of age at diagnosis and ER status for the first breast cancer showed significant declines in SIRs among all age groups except for age < 40 years and some variability by ER status with significant trends only observed among ER-positive patients (Additional file 1: Supplementary Fig. 1).

Overall significant decreasing trends in SIRs remained when analyses were restricted to < 5 years since first diagnosis with trends even more pronounced among women aged < 50 years at first diagnosis and those diagnosed with a first ER-negative tumor (Additional file 1: Supplementary Table 2). Significant declines in risk were also observed when analyses included women diagnosed with in situ CBC (Additional file 1: Supplementary Fig. 2). Trends in SIRs were similar when we varied the definition of CBC to those diagnosed ≥ 6 or ≥ 24 months after the first breast cancer (data not shown). Exploratory analyses suggest that CBC risk declined over time among women treated with chemotherapy, particularly among women diagnosed with a first breast cancer prior to age 50 years (Additional file 1: Supplementary Fig. 3), and among women treated with hormone therapy (Additional file 1: Supplementary Fig. 4).

Table 3 shows estimated 5-year cumulative incidence of CBC in women diagnosed with a first breast cancer in the recent treatment era (2004 to 2015 and followed through 2016). Breast cancer patients had an overall 5-year cumulative incidence of CBC of 1.31% (95% CI = 1.26–1.37%). The highest 5-year cumulative incidence was observed after a first ER-negative tumor in women aged < 50 years at diagnosis (1.89%, 95% CI = 1.61–2.21%) and lowest after an ER-positive tumor in women aged < 50 years at diagnosis (1.01%, 95% CI = 0.90–1.14%). In a subgroup of women diagnosed with a first breast cancer between 2010 and 2015 (follow-up through 2016), the 5-year cumulative incidence did not differ by HER2 status and the highest cumulative incidence was observed after a first triple-negative breast tumor (1.71%, 95% CI = 1.38–2.09%). Estimates of cumulative incidence were overall similar, but slightly higher, among 6-month survivors (Additional file 1: Supplementary Table 3).

**Table 3:** Cumulative incidence of contralateral breast cancer among 1-year survivors of a first primary breast cancer diagnosed in the recent treatment era between 2004 and 2015 (followed through to 2016) in 12 SEER registries

First breast cancer characteristic	5-year cumulative incidence (95% CIs)^a^
Overall	1.31% (1.26 to 1.37%)
Age at diagnosis, years	
< 40	1.54% (1.27 to 1.85%)
40 to < 50	1.15% (1.03 to 1.28%)
50 to < 60	1.24% (1.13 to 1.35%)
60 to < 70	1.33% (1.22 to 1.45%)
≥ 70	1.47% (1.35 to 1.60%)
Stage at diagnosis	
I	1.32% (1.24 to 1.40%)
II	1.17% (1.08 to 1.26%)
III	1.73% (1.55 to 1.93%)
ER status	
Positive	1.19% (1.13 to 1.26%)
Negative	1.81% (1.66 to 1.98%)
ER status and age at diagnosis	
ER-positive	
< 50 years	1.01% (0.90 to 1.14%)
≥ 50 years	1.24% (1.17 to 1.32%)
ER-negative	
< 50 years	1.89% (1.61 to 2.21%)
≥ 50 years	1.78% (1.60 to 1.97%)
HER2-status^b^	
Positive	1.35% (1.05 to 1.71%)
Negative	1.33% (1.21 to 1.45%)
Breast cancer subtype^b,c^	
HR+/HER2+	1.39% (1.02 to 1.84%)
HR+/HER2−	1.27% (1.15 to 1.41%)
HR−/HER2+	1.27% (0.80 to 1.94%)
HR-/HER2- (triple negative)	1.71% (1.38 to 2.09%)

The recent treatment era included 198,496 women diagnosed with a first primary breast cancer between 2004 and 2015 and 3179 CBC cases developed through 2016. Women with contralateral prophylactic mastectomies or unknown type of surgery were excluded from all estimates of cumulative incidence

*SEER*, Surveillance, Epidemiology, and End Results; *CI*, confidence interval; *ER*, estrogen-receptor; *HER2*, human epidermal growth factor receptor 2; *HR*, hormone receptor; *PR*, progesterone receptor

^a^Cumulative incidence was calculated from the index date (the first 12 months after the initial breast cancer diagnosis were excluded)

^b^HER2-status and breast cancer subtype were estimated in a subgroup of women diagnosed with a first cancer between 2010 and 2015 and followed through 2016 (*N* = 100,153; CBC = 762; overall 5-year cumulative incidence = 1.34% (1.23–1.45%)

^c^HR+ = ER+ and/or PR+; HR− = ER− and PR−

## Discussion

In this large population-based study from 1992 to 2016, we found that breast cancer patients had approximately twice the risk of an incident breast cancer (in the contralateral breast) when compared to that expected in the general population. However, our analysis showed a decline in CBC risk over calendar time, consistent with advances in breast cancer treatment, particularly after a first ER-positive breast cancer. Notably, the 5-year cumulative incidence of CBC was low, ranging from 1 to 2%, among women diagnosed with a first breast cancer during the most recent treatment era.

Previous studies examining trends in CBC incidence among US women have reported a decline since the early 1980s through 2006 [3, 4]. Our study extended these findings by 10 years into the modern treatment era and expanded results by examining CBC risk according to characteristics of the first breast cancer, including HER2 status and IHC-defined subtype. In addition, we extended previous findings by calculating SIRs to examine trends in CBC risk relative to the general population. The calculation of SIRs extracts the effect of treatment from broader changes in breast cancer risk factors (e.g., parity, hormone use, and obesity) which may impact both first and second breast cancer risk. Furthermore, we were able to examine the effect of contralateral prophylactic mastectomies on temporal trends in CBC risk. Our finding that declines in CBC risk remained even with the exclusion of women with contralateral prophylactic mastectomies suggests that decreases in CBC risk are likely due to advances in systemic therapies.

In our study, we observed that CBC risk after an ER-positive tumor has continued to decline past 2006, the end of our previous follow-up period [4]. The widespread introduction of aromatase inhibitors in the mid-2000s likely contributed to this continuing decline among older women (≥ 50 years) as randomized clinical trials have shown a greater reduction in CBC with aromatase inhibitors as compared to tamoxifen [21]. In younger ER-positive women (< 50 years), we found that the decline in CBC risk was not completely driven by the increase in prophylactic mastectomies, which suggests a continuing impact of the widespread use of tamoxifen [22, 23] and possibly changes in chemotherapy regimens [6, 7].

Interestingly, we observed an overall decline in CBC risk after an ER-negative tumor which differs from previously reported trends through 2006, where there were no clear decreases in CBC incidence after an ER-negative breast cancer [4]. In our study, the observed decline in CBC risk after an ER-negative tumor appeared among women diagnosed with a first breast cancer in 2004 to 2009 with a further decline among those diagnosed between 2010 and 2015, particularly among those aged < 50 years. Further advances in chemotherapy, such as a shift towards taxane-based chemotherapy in the mid-2000s, might have partially driven the observed decline in CBC risk after an ER-negative tumor [6, 7]. A recent study conducted among Dutch women diagnosed with a first breast cancer between 2003 and 2010 found that taxane-containing chemotherapy was associated with a strong reduction in CBC risk compared to no chemotherapy (HR = 0.48, 95% CI = 0.36–0.62) [6]. Further studies are needed to confirm these findings and to elucidate the potential role of chemotherapy regimens on CBC risk.

In our study, we observed a 5-year cumulative incidence of CBC of 1.31% for women diagnosed with a first breast cancer in the recent treatment era. These results are substantially lower than earlier studies [24–26]. However, previous studies included women diagnosed with a first breast cancer between the 1930s and mid-1990s, and thus, largely prior to adjuvant hormone treatments and modern chemotherapy, and possibly also when the proportion of ER-negative tumors was higher [27]. Our 5-year cumulative incidence estimates, however, are consistent with more recent studies from other countries with similar treatment patterns [6, 28–30].

This is the first study to our knowledge to provide population-based estimates of 5-year cumulative incidence of CBC in US breast cancer patients according to HER2 status and breast cancer subtype, which only became available in the SEER registries starting in 2010. Although we found no difference in the 5-year cumulative incidence by HER2 status of the first tumor, we observed the highest 5-year cumulative incidence of CBC after a triple-negative tumor. These results differ slightly from the previous Dutch study [6] which reported a higher 5-year cumulative incidence of CBC after HER2-negative disease (1.9%, 95% CI = 1.8–2.0%) compared to HER2-positive disease (1.5%, 95% CI = 1.3–1.7%). To date, clinical trial data in women with early stage HER2-positive breast cancer have shown no difference in CBC incidence among those treated with versus without trastuzumab [31]. Future studies with long-term follow-up are needed to examine the impact of treatment on CBC risk in early-stage HER2-positive patients.

The strengths of this study include the large-scale and population-based design with almost 13,000 CBCs, long-term follow-up from 1992 to 2016, and detailed tumor characteristics, which provided the opportunity to assess CBC risk and temporal trends in risk by characteristics of the first breast cancer. In addition, we had surgical data on the removal of the uninvolved contralateral breast and were therefore able to describe the overall limited impact of contralateral prophylactic mastectomies on the declining trends in CBC risk. There were also several limitations to our analysis including lack of detailed treatment data and individual-level risk factors. Due to under ascertainment of cancer treatment in the SEER registries, we were unable to directly compare CBC risk among women who did and did not receive treatment. Importantly, specificity of treatment data in SEER registries is high and therefore it is unlikely that known receipt of treatment is misclassified [15]. In addition, although we assumed that CBC was not a metastatic lesion or local recurrence of the original breast cancer, it is possible that CBC was misclassified [32–34]. However, we excluded the first 12 months of follow-up to reduce this potential misclassification. Furthermore, our results were similar when we varied the criteria to define CBC as those occurring ≥ 6 or 24 months after the first breast cancer.

## Conclusions

In summary, the decline in CBC risk in breast cancer patients is likely related to continued advances in systemic breast cancer treatment. Although breast cancer patients had an overall increased risk of developing an incident breast cancer (in the contralateral breast) when compared to that expected in the general population, the current probability of developing CBC at 5 years was low (1–2%). These estimates can further inform breast cancer patients and clinicians on the risk of CBC and help guide treatment decisions.

## Supplementary Information

**Additional file 1.** Contains supplementary tables and figures.

## Data Availability

The datasets analyzed during the current study are available from the National Cancer Institute’s Surveillance, Epidemiology and End Results program database: https://seer.cancer.gov/data/access.html

## Abbreviations

CBC: Contralateral breast cancer
SIR: Standardized incidence ratio
CI: Confidence interval
ER: Estrogen receptor
HER2: Human epidermal growth factor receptor 2
HR: Hormone Receptor
IHC: Immunohistochemistry
