# Supplementary material for: Risk of contralateral breast cancer according to first breast cancer characteristics among women in the USA, 1992–2016
Source: Breast Cancer Res. 2021 Feb 17;23:24. doi: 10.1186/s13058-021-01400-3 (PMC7890613; doi:10.1186/s13058-021-01400-3)

**Additional File 1: Supplementary Tables and Figures**

**Supplementary Table 1.** Temporal trends in SIRs for contralateral breast cancer among 1-year survivors of a first primary breast cancer in 12 SEER registries, 1992-2016

**Supplementary Table 2.** Temporal trends in SIRs for contralateral breast cancer by latency among 1-year survivors of a first primary breast cancer in 12 SEER registries, 1992-2016

**Supplementary Table 3.** Cumulative incidence of contralateral breast cancer among 6-month survivors of a first primary breast cancer diagnosed in the recent treatment era between 2004-2015 (followed through 2016) in 12 SEER registries

**Supplementary Figure 1.** Temporal trends in SIRs for contralateral breast cancer according to detailed categories of age at diagnosis among 1-year survivors of a first primary breast cancer in 12 SEER registries, 1992-2016.

**Supplementary Figure 2.** Temporal trends in SIRs for invasive contralateral breast cancer only and both *in situ* + invasive contralateral breast cancer among 1-year survivors of a first primary breast cancer in 12 SEER registries, 1992-2016

**Supplementary Figure 3.** Temporal trends in SIRs for contralateral breast cancer by initial chemotherapy (yes and no/unknown) among 1-year survivors of a first primary breast cancer in 12 SEER registries, 1992-2016

**Supplementary Figure 4.** Temporal trends in SIRs for contralateral breast cancer by initial hormone therapy (yes and no/unknown) among 1-year survivors of a first primary breast cancer in 12 SEER registries, 1992-2016

| **Supplementary Table 1.** Temporal trends in SIRs for contralateral breast cancer among 1-year survivors of a first primary breast cancer in 12 SEER registries, 1992-2016 | | | | | | | | | | | |
| --- | --- | --- | --- | --- | --- | --- | --- | --- | --- | --- | --- |
|  | Calendar year of first breast cancer diagnosis | | | | | | | | |  |  |
|  | 1992-1997 | | 1998-2003 | | 2004-2009 | | 2010-2015 | |  | | *Ptrend* |
| First breast cancer characteristic | No. | SIR (95% CI) | No. | SIR (95% CI) | No. | SIR (95% CI) | No. | SIR (95% CI) |  | |  |
| **Overall** |  |  |  |  |  |  |  |  |  | |  |
| All | 5135 | 2.49 (2.42 to 2.56) | 4582 | 2.28 (2.21 to 2.35) | 2483 | 1.90 (1.83 to 1.98) | 786 | 1.57 (1.46 to 1.68) |  | | <0.001 |
| Excluding CPM^a^ | -- | -- | 4369 | 2.34 (2.27 to 2.41) | 2417 | 2.00 (1.92 to 2.08) | 762 | 1.71 (1.59 to 1.84) |  | | <0.001 |
| **Age at diagnosis** |  |  |  |  |  |  |  |  |  | |  |
| <50 years |  |  |  |  |  |  |  |  |  | |  |
| All | 1599 | 3.48 (3.31 to 3.65) | 1317 | 3.53 (3.34 to 3.73) | 636 | 2.86 (2.64 to 3.09) | 141 | 2.11 (1.78 to 2.49) |  | | <0.001 |
| Excluding CPM^a^ | -- | -- | 1248 | 3.71 (3.51 to 3.92) | 616 | 3.18 (2.93 to 3.44) | 136 | 2.66 (2.23 to 3.15) |  | | <0.001 |
| ≥50 years |  |  |  |  |  |  |  |  |  | |  |
| All | 3536 | 2.21 (2.14 to 2.28) | 3265 | 2.00 (1.93 to 2.07) | 1847 | 1.70 (1.62 to 1.78) | 645 | 1.49 (1.38 to 1.61) |  | | <0.001 |
| Excluding CPM^a^ | -- | -- | 3121 | 2.04 (1.97 to 2.11) | 1801 | 1.77 (1.69 to 1.85) | 626 | 1.59 (1.47 to 1.72) |  | | <0.001 |
| **ER status** |  |  |  |  |  |  |  |  |  | |  |
| Positive |  |  |  |  |  |  |  |  |  | |  |
| All | 3065 | 2.31 (2.23 to 2.39) | 2952 | 2.05 (1.98 to 2.13) | 1734 | 1.68 (1.60 to 1.76) | 621 | 1.47 (1.36 to 1.59) |  | | <0.001 |
| Excluding CPM^a^ | -- | -- | 2840 | 2.12 (2.04 to 2.20) | 1686 | 1.76 (1.68 to 1.85) | 602 | 1.59 (1.47 to 1.72) |  | | <0.001 |
| Negative |  |  |  |  |  |  |  |  |  | |  |
| All | 1166 | 3.12 (2.94 to 3.30) | 1054 | 3.32 (3.12 to 3.53) | 651 | 2.86 (2.64 to 3.09) | 153 | 2.14 (1.81 to 2.51) |  | | <0.001 |
| Excluding CPM^a^ | -- | -- | 994 | 3.37 (3.16 to 3.59) | 634 | 3.03 (2.80 to 3.28) | 148 | 2.41 (2.04 to 2.83) |  | | <0.001 |
| **ER-status and age at diagnosis** |  |  |  |  |  |  |  |  |  | |  |
| Positive |  |  |  |  |  |  |  |  |  | |  |
| <50 years |  |  |  |  |  |  |  |  |  | |  |
| All | 750 | 2.87 (2.67 to 3.08) | 684 | 2.80 (2.59 to 3.02) | 384 | 2.29 (2.07 to 2.53) | 106 | 1.95 (1.60 to 2.36) |  | | <0.001 |
| Excluding CPM^a^ | -- | -- | 656 | 2.98 (2.76 to 3.22) | 370 | 2.55 (2.30 to 2.82) | 101 | 2.41 (1.96 to 2.93) |  | | 0.006 |
| ≥50 years |  |  |  |  |  |  |  |  |  | |  |
| All | 2315 | 2.18 (2.09 to 2.27) | 2268 | 1.90 (1.82 to 1.98) | 1350 | 1.56 (1.48 to 1.65) | 515 | 1.40 (1.28 to 1.53) |  | | <0.001 |
| Excluding CPM^a^ | -- | -- | 2184 | 1.95 (1.87 to 2.03) | 1316 | 1.62 (1.53 to 1.71) | 501 | 1.49 (1.36 to 1.63) |  | | <0.001 |
| Negative |  |  |  |  |  |  |  |  |  | |  |
| <50 years |  |  |  |  |  |  |  |  |  | |  |
| All | 569 | 4.78 (4.40 to 5.19) | 451 | 5.48 (4.99 to 6.01) | 228 | 4.85 (4.24 to 5.52) | 32 | 2.73 (1.87 to 3.85) |  | | 0.23 |
| Excluding CPM^a^ | -- | -- | 425 | 5.71 (5.18 to 6.28) | 222 | 5.44 (4.75 to 6.20) | 32 | 3.81 (2.61 to 5.38) |  | | 0.06 |
| ≥50 years |  |  |  |  |  |  |  |  |  | |  |
| All | 597 | 2.34 (2.16 to 2.54) | 603 | 2.56 (2.36 to 2.77) | 423 | 2.34 (2.12 to 2.57) | 121 | 2.02 (1.68 to 2.41) |  | | 0.31 |
| Excluding CPM^a^ | -- | -- | 569 | 2.58 (2.37 to 2.80) | 412 | 2.45 (2.22 to 2.70) | 116 | 2.19 (1.81 to 2.63) |  | | 0.10 |
| Abbreviations: SEER, Surveillance, Epidemiology, and End Results; SIRs, standardized incidence ratio; CI, confidence interval; ER, estrogen receptor; CPM, contralateral prophylactic mastectomy  ^a^ Women with contralateral prophylactic mastectomy or unknown type of surgery were excluded | | | | | | | | | | | |

| **Supplementary Table 2.** Temporal trends in SIRs for contralateral breast cancer by latency among 1-year survivors of a first primary breast cancer in 12 SEER registries, 1992-2016 | | | | | | | | | | |
| --- | --- | --- | --- | --- | --- | --- | --- | --- | --- | --- |
|  | Calendar year of first breast cancer diagnosis | | | | | | | |  |  |
|  | 1992-1997 | | 1998-2003 | | 2004-2009 | | 2010-2015 | |  | *Ptrend* |
| First breast cancer characteristic | No. | SIR (95% CI) | No. | SIR (95% CI) | No. | SIR (95% CI) | No. | SIR (95% CI) |  |  |
| **<5 years after first cancer diagnosis** |  |  |  |  |  |  |  |  |  |  |
| Overall | 1442 | 2.61 (2.48 to 2.75) | 1469 | 2.30 (2.18 to 2.42) | 1022 | 1.63 (1.53 to 1.73) | 687 | 1.52 (1.41 to 1.64) |  | <0.001 |
| Age at diagnosis |  |  |  |  |  |  |  |  |  |  |
| <50 years | 410 | 5.19 (4.70 to 5.72) | 411 | 4.45 (4.03 to 4.90) | 255 | 2.66 (2.34 to 3.01) | 127 | 2.14 (1.78 to 2.55) |  | <0.001 |
| ≥50 years | 1032 | 2.18 (2.05 to 2.32) | 1058 | 1.93 (1.82 to 2.05) | 767 | 1.45 (1.35 to 1.56) | 560 | 1.43 (1.31 to 1.55) |  | <0.001 |
| ER status |  |  |  |  |  |  |  |  |  |  |
| Positive | 819 | 2.26 (2.11 to 2.42) | 879 | 1.91 (1.79 to 2.04) | 682 | 1.38 (1.28 to 1.49) | 544 | 1.43 (1.31 to 1.56) |  | <0.001 |
| Negative | 353 | 3.83 (3.44 to 4.25) | 386 | 3.88 (3.50 to 4.29) | 299 | 2.76 (2.46 to 3.09) | 134 | 2.07 (1.73 to 2.45) |  | <0.001 |
| ER-status and age at diagnosis |  |  |  |  |  |  |  |  |  |  |
| Positive |  |  |  |  |  |  |  |  |  |  |
| <50 years | 174 | 3.85 (3.30 to 4.47) | 190 | 3.16 (2.73 to 3.64) | 138 | 1.91 (1.60 to 2.26) | 97 | 2.02 (1.64 to 2.46) |  | <0.001 |
| ≥50 years | 645 | 2.03 (1.88 to 2.19) | 689 | 1.73 (1.60 to 1.86) | 544 | 1.29 (1.18 to 1.40) | 447 | 1.34 (1.22 to 1.47) |  | <0.001 |
| Negative |  |  |  |  |  |  |  |  |  |  |
| <50 years | 154 | 7.61 (6.46 to 8.91) | 160 | 7.79 (6.63 to 9.10) | 106 | 5.27 (4.31 to 6.37) | 28 | 2.68 (1.78 to 3.87) |  | <0.001 |
| ≥50 years | 199 | 2.76 (2.39 to 3.17) | 226 | 2.86 (2.50 to 3.26) | 193 | 2.18 (1.88 to 2.51) | 106 | 1.96 (1.60 to 2.37) |  | <0.001 |
| **≥5 years after first cancer diagnosis** |  |  |  |  |  |  |  |  |  |  |
| Overall | 3693 | 2.45 (2.37 to 2.53) | 3113 | 2.28 (2.20 to 2.36) | 1461 | 2.14 (2.03 to 2.25) | 99 | 2.02 (1.64 to 2.46) |  | <0.001 |
| Age at diagnosis |  |  |  |  |  |  |  |  |  |  |
| <50 years | 1189 | 3.13 (2.95 to 3.31) | 906 | 3.23 (3.02 to 3.45) | 381 | 3.00 (2.71 to 3.32) | 14 | 1.83 (1.00 to 3.07) |  | 0.35 |
| ≥50 years | 2504 | 2.22 (2.13 to 2.31) | 2207 | 2.03 (1.95 to 2.12) | 1080 | 1.94 (1.83 to 2.06) | 85 | 2.05 (1.64 to 2.53) |  | <0.001 |
| ER status |  |  |  |  |  |  |  |  |  |  |
| Positive | 2246 | 2.33 (2.23 to 2.43) | 2073 | 2.12 (2.03 to 2.21) | 1052 | 1.96 (1.84 to 2.08) | 77 | 1.86 (1.47 to 2.32) |  | <0.001 |
| Negative | 813 | 2.89 (2.69 to 3.10) | 668 | 3.06 (2.83 to 3.30) | 352 | 2.94 (2.64 to 3.26) | 19 | 2.75 (1.65 to 4.29) |  | 0.65 |
| ER-status and age at  diagnosis |  |  |  |  |  |  |  |  |  |  |
| Positive |  |  |  |  |  |  |  |  |  |  |
| <50 years | 576 | 2.67 (2.46 to 2.90) | 494 | 2.68 (2.45 to 2.93) | 246 | 2.59 (2.28 to 2.93) | 9 | 1.44 (0.66 to 2.73) |  | 0.37 |
| ≥50 years | 1670 | 2.24 (2.13 to 2.35) | 1579 | 1.99 (1.89 to 2.09) | 806 | 1.83 (1.71 to 1.96) | 68 | 1.94 (1.51 to 2.46) |  | <0.001 |
| Negative |  |  |  |  |  |  |  |  |  |  |
| <50 years | 415 | 4.21 (3.81 to 4.64) | 291 | 4.71 (4.18 to 5.28) | 122 | 4.54 (3.77 to 5.42) | 4 | 3.13 (0.84 to 8.01) |  | 0.35 |
| ≥50 years | 398 | 2.18 (1.97 to 2.41) | 377 | 2.41 (2.17 to 2.67) | 230 | 2.48 (2.17 to 2.82) | 15 | 2.66 (1.49 to 4.39) |  | 0.07 |
| Abbreviations: SEER, Surveillance, Epidemiology, and End Results; SIRs, standardized incidence ratio; CI, confidence interval; ER, estrogen receptor | | | | | | | | | | |

| **Supplementary Table 3.** Cumulative incidence of contralateral breast cancer among 6-month survivors of a first primary breast cancer diagnosed in the recent treatment era between 2004-2015 (followed through 2016) in 12 SEER registries^a,b,c^ | |
| --- | --- |
| First breast cancer characteristic | 5-year cumulative incidence (95% CIs)^d^ |
| Overall | 1.41% (1.35% to 1.47%) |
| Age at diagnosis, years |  |
| <40 | 1.63% (1.36% to 1.95%) |
| 40-<50 | 1.23% (1.11% to 1.36%) |
| 50-<60 | 1.36% (1.25% to 1.48%) |
| 60-<70 | 1.44% (1.32% to 1.56%) |
| ≥70 | 1.55% (1.43% to 1.68%) |
| Stage |  |
| I | 1.41% (1.33% to 1.49%) |
| II | 1.25% (1.16% to 1.35%) |
| III | 1.92% (1.73% to 2.12%) |
| ER status |  |
| Positive | 1.30% (1.23% to 1.36%) |
| Negative | 1.89% (1.73% to 2.05%) |
| ER status and age at diagnosis |  |
| ER-positive |  |
| <50 years | 1.11% (0.99% to 1.24%) |
| ≥50 years | 1.35% (1.28% to 1.43%) |
| ER-negative |  |
| <50 years | 1.93% (1.65% to 2.25%) |
| ≥50 years | 1.87% (1.69% to 2.06%) |
| HER2-status^d^ |  |
| Positive | 1.36% (1.07% to 1.70%) |
| Negative | 1.43% (1.32% to 1.56%) |
| Breast cancer subtype^e,f^ |  |
| HR+/HER2+ | 1.38% (1.04% to 1.80%) |
| HR+/ HER2- | 1.38% (1.25% to 1.51%) |
| HR-/ HER2+ | 1.33% (0.85% to 1.98%) |
| Triple negative | 1.83% (1.50% to 2.21%) |
| Abbreviations: SEER, Surveillance, Epidemiology, and End Results; CI, confidence interval; ER, estrogen-receptor; HER2, human epidermal growth factor receptor 2; HR, hormone receptor; PR, progesterone receptor | |
| ^a^ CBC was defined as a second invasive breast cancer in the contralateral breast ≥6 months after the first breast cancer. | |
| ^b^ The recent treatment era included 203,120 women diagnosed with a first primary breast cancer between 2004 to 2015 and 3,421 CBC cases developed through 2016. | |
| ^c^ Women with contralateral prophylactic mastectomies or unknown surgery type were excluded. | |
| ^d^ Cumulative incidence was calculated from the index date (the first 6 months after the initial breast cancer diagnosis were excluded). | |
| ^e^ HER2-status and subtype were estimated in a subgroup of women diagnosed with a first cancer between 2010-2015 and followed through 2016 (N=103,288 ; CBC=889; overall 5-year cumulative incidence=1.43% [1.32%-1.54%]). | |
| ^f^ HR+ = ER+ and/or PR+; HR- = ER- and PR-. | |

**Supplementary Figure 1.** Temporal trends in SIRs for contralateral breast cancer according to detailed categories of age at diagnosis among 1-year survivors of a first primary breast cancer in 12 SEER registries, 1992-2016. Estimates for contralateral breast cancer by calendar period of first breast cancer diagnosis are according to (A) age at diagnosis, (B) after ER-positive breast cancer by age at first diagnosis, and (C) after ER-negative breast cancer by age at first diagnosis.


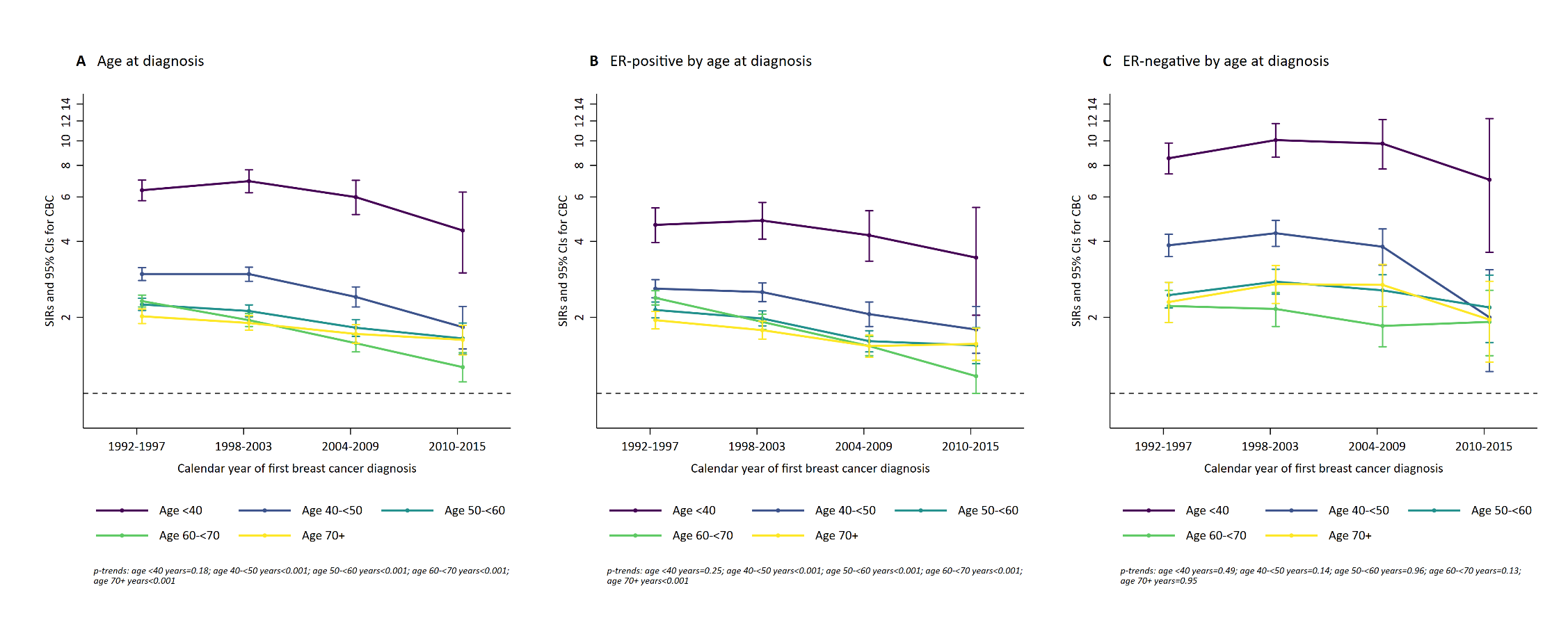


**Supplementary Figure 2.** Temporal trends in SIRs for invasive contralateral breast cancer (CBC) only and both *in situ* + invasive CBC among 1-year survivors of a first primary breast cancer in 12 SEER registries, 1992-2016. Estimates for CBC by calendar year of first breast cancer diagnosis are (A) overall, and according to (B-C) age at first breast cancer diagnosis, (D-E) ER status of first breast cancer, (F-G) after ER-positive breast cancer by age at first diagnosis, and (H-I) after ER-negative breast cancer by age at first diagnosis.


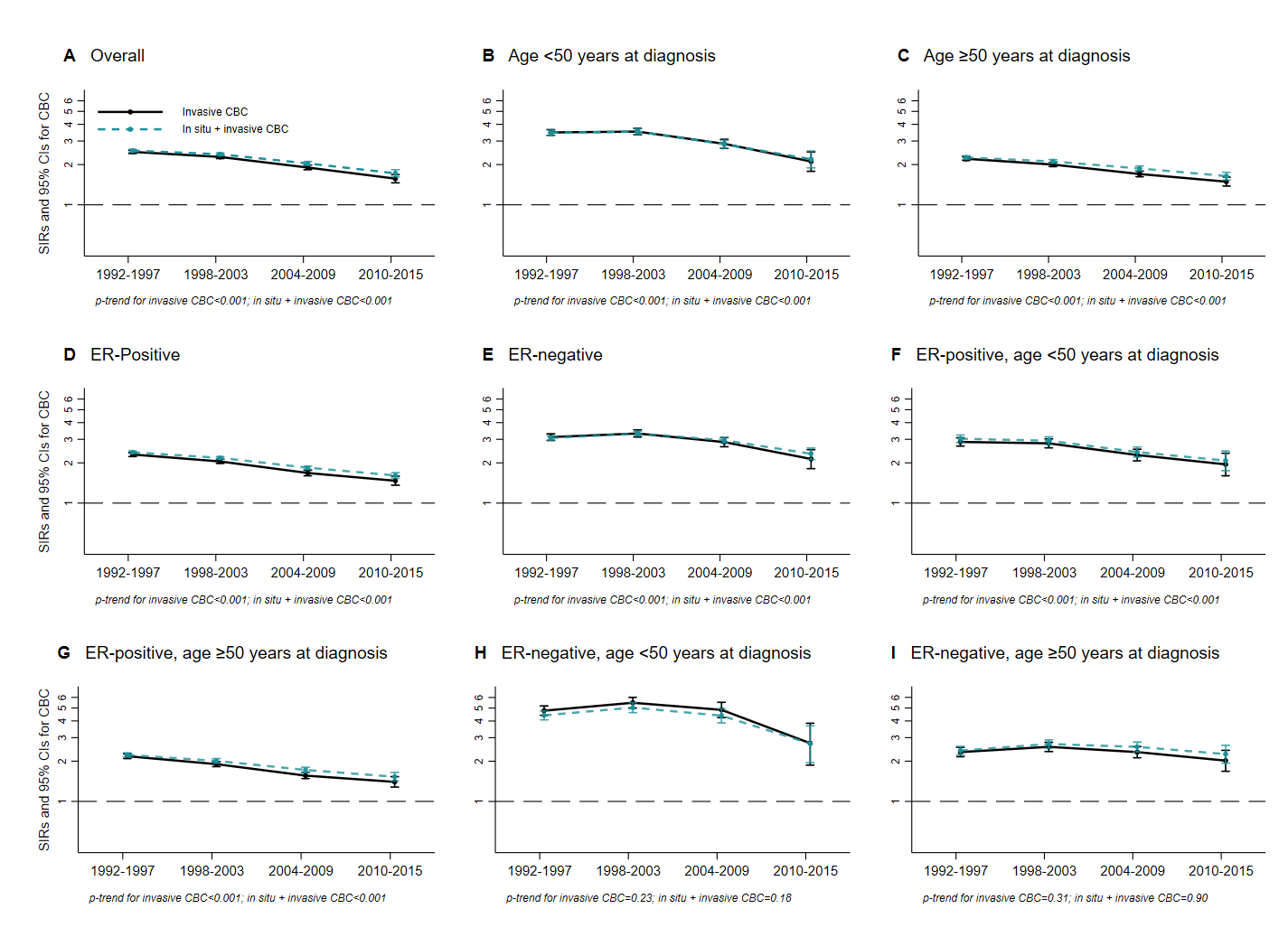


**Supplementary Figure 3.** Temporal trends in SIRs for contralateral breast cancer (CBC) by initial chemotherapy (yes and no/unknown) among 1-year survivors of a first primary breast cancer in 12 SEER registries, 1992-2016. Estimates for CBC by calendar year of first breast cancer diagnosis and initial chemotherapy are (A) overall, and according to (B-C) age at first breast cancer diagnosis, (D-E) ER status of first breast cancer, (F-G) after ER-positive breast cancer by age at first diagnosis, and (H-I) after ER-negative breast cancer by age at first diagnosis.


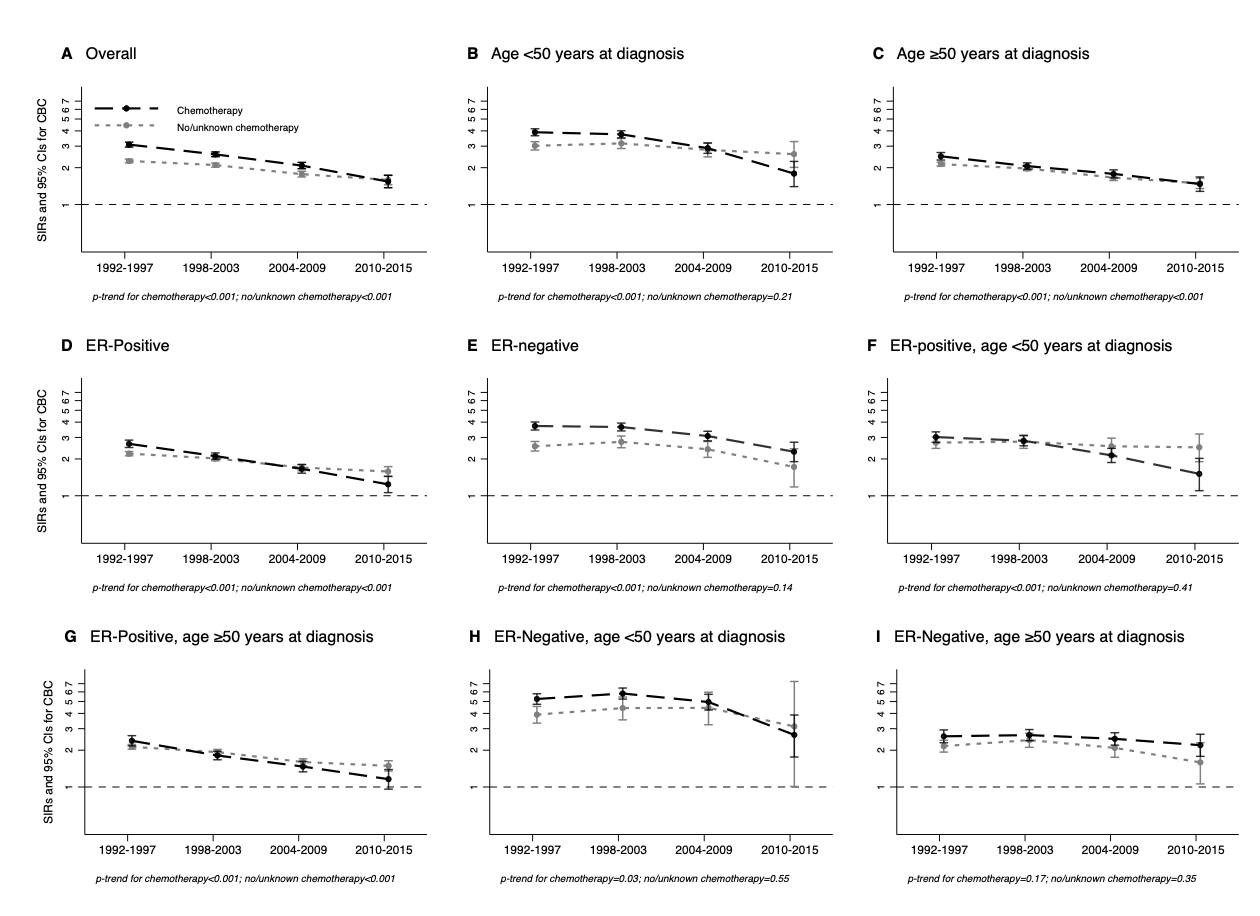


**Supplementary Figure 4.** Temporal trends in SIRs for contralateral breast cancer (CBC) by initial hormone therapy (yes and no/unknown) among 1-year survivors of a first primary breast cancer in 12 SEER registries, 1992-2016. Estimates for CBC by calendar year of first breast cancer diagnosis and initial chemotherapy are (A) overall, and according to (B-C) age at first breast cancer diagnosis, (D-E) ER status of first breast cancer, (F-G) after ER-positive breast cancer by age at first diagnosis.


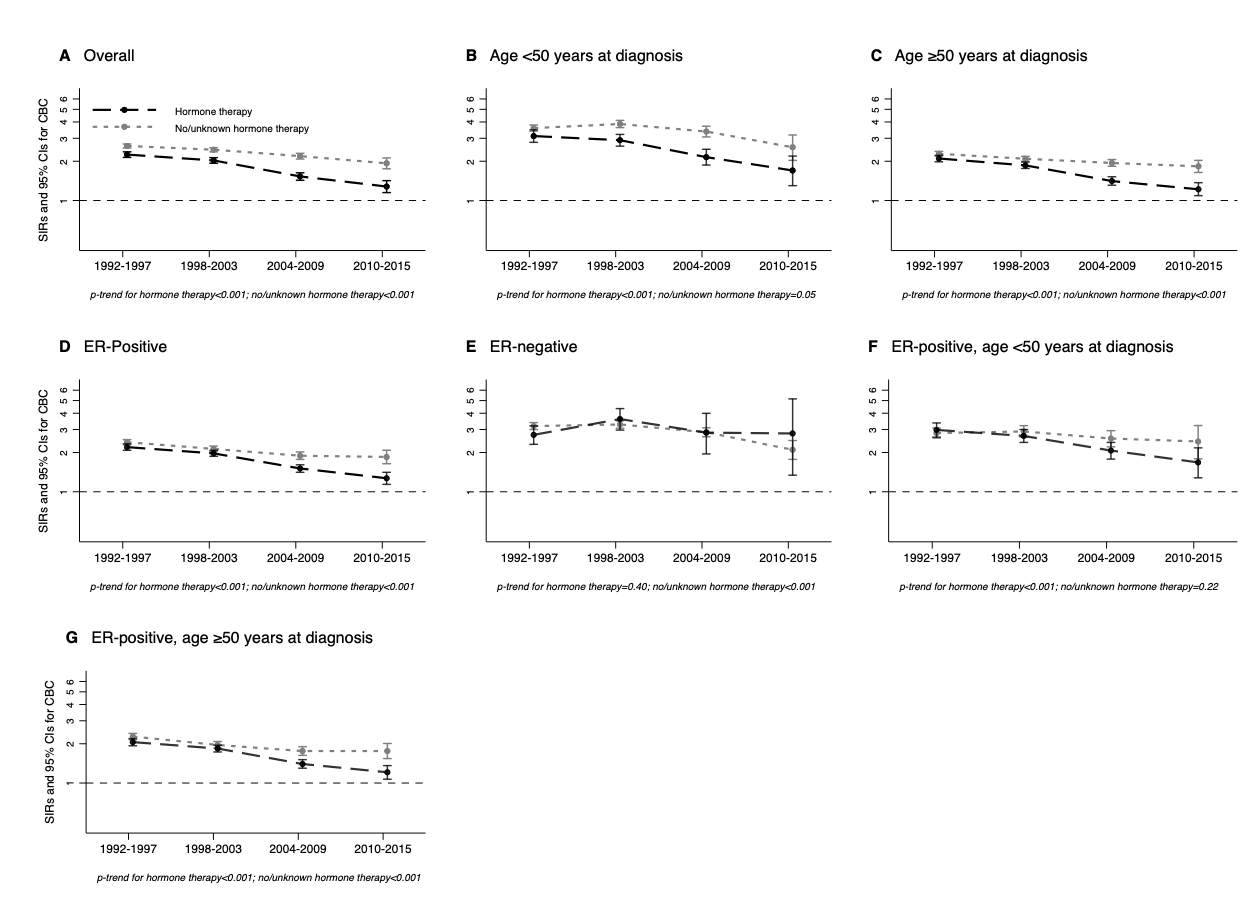

Supplement: Supplementary file 1 — Additional file 1. Contains supplementary tables and figures. [file 13058_2021_1400_MOESM1_ESM.docx]
